# Sponsored by the State: The Private Regulation of Government Influencers

**DOI:** 10.1007/s10603-025-09598-x

**Published:** 2025-09-16

**Authors:** Taylor Annabell, Catalina Goanta, Thijs Kelder, Felix Pflücke

**Affiliations:** 1 Molengraaff Institute for Private Law, Utrecht University, Utrecht, Netherlands; 2 Department of Law, University of Luxembourg, Luxembourg, Luxembourg; 3 Faculty of Law, University of Oxford, Oxford, United Kingdom

**Keywords:** Influencer marketing, Government communication, Political advertising, Transparency, Consumer law and policy

## Abstract

The popularity of influencer marketing is ever growing. Based on parasocial relationships rooted in authenticity and relatability, the appeal of influencers is effectively used to promote commercial goods and services. This popularity is increasingly migrating outside of commercial advertising. In the past years, governments around the world have collaborated with influencers for public interest communication such as supporting wars, promoting Covid public health policies or financial literacy. Although entrenched in promoting the public good and facilitated by public funding, the dynamics of these collaborations remain very much unknown. Shedding light on how governments employ influencers can help us understand how commercial strategies shape the advertising of public goods as state propaganda. From a regulatory perspective, commercial advertising has been subject to a lot of rules relating to the content as well as the transparency of commercial messaging. Yet government communication—whether called propaganda, public service communication, or the advertising of public goods—has not been governed with the same level of clarity. This study explores comprehensive materials from 10 freedom of information requests on government influencer campaigns answered by the Dutch government between 2020 and 2024 (*N* = 1302 pages). Using qualitative content analysis, we focus on understanding the characteristics of advertising contracts between the government and the influencers in their service, in order to critically reflect on the transparency of the monetisation entailed by these transactions.

## Introduction

The year is 2024. A Gen Z TikTok user opens the app. The screen turns green from a niche meme used by Kamala Harris’ social media strategists during her presidential campaign. A few more swipe ups reveal videos of different creators chiming in on the value of this meme or scenes from podcasts where the discussion revolves around abortion rights, freeing Palestine, or trad wives. More swipe ups bring Vine-like videos of politicians such as Dutch member of parliament Thierry Baudet engaging in thirst-traps to convince his followers to get out and vote for the national elections. Such a “For You Page” on a popular social media app is not necessarily the result of a well-curated source of information for a politically minded user. Such content has rather become commonplace, reflecting the demand—namely the interest of newer (and older) generations in political engagement and empowerment—and supply—content creation in various forms and by various stakeholders—of politics on social media (Salte, 2022).

This reality can be partially explained by the popularity of influencers. The rise of social media influencers has revolutionised marketing, creating a dynamic landscape where individuals with significant online followings can shape consumer behaviour and public opinion, and can have more consumer impact than brand-only advertising (Lee et al., 2024). This transformation extends beyond commercial marketing into the realm of political and public sector communication, where governments now employ influencers to disseminate information and influence public attitudes. Influencers are increasingly used in the political arena not only to support electoral candidates and their policies but also to promote propaganda during war, health (mis)information during pandemics, or engage new audiences in the Olympic Games (New York Times, 2024). So much so, that even political influencer marketing agencies have started to emerge around the world. While a lot of attention has been given particularly to the “politicisation” of influencers, a lot of issues remain unmapped. One of these issues pertains to the legal regimes applicable to government influencers, namely influencers hired to perform influencer marketing on behalf of different state institutions. Caught between laws, contracts and platform policies, government influencers lend their voice to public interest advertising, or propaganda—as the case may be. Government influencers find themselves in a tug of war between their individual freedom of expression and the commercial underpinnings of their personal brand. Yet as attractive as it is to employ influencers to persuade audiences about government messaging, these collaborations have their own vulnerabilities. On the one hand, influencers may change their mind about supporting certain policies, and on the other hand, governments may depend on the feeble reputation of influencers, who can be cancelled or deplatformed overnight.

To better understand this landscape, our paper examines the regulation of government influencers, focusing on legal frameworks and contractual dynamics, particularly within the European Union (EU) and the Netherlands. Overall, the paper highlights that current advertising regulation does not accurately govern the phenomenon of government influencers, and it empirically shows the commercial and transactional nature of the campaigns where the government works with influencers. As a result, we put forth the argument that such campaigns should be treated as commercial undertakings, and be governed by existing regulation in the field. The paper is structured as follows. Section 2 argues that social media influencers are subject to legal frameworks like the Unfair Commercial Practices Directive (UCPD) and the Political Advertising Regulation (PAR), which mandate transparency in sponsored content to prevent consumer and citizen deception. Section 3 outlines the legal qualification for government influencers. Section 4 contains an empirical study on Dutch government influencers, utilising data from Freedom of Information (FoI) requests to analyse influencer campaigns across different ministries, revealing varying conceptualisations and strategic uses of influencers within governmental contexts. Section 5 explores the contractual dynamics between Dutch government entities and influencers, highlighting the legal implications of these agreements and influencers’ roles as natural or legal persons under consumer law. Section 6 draws lessons from EU consumer law and policy to outline the dangers of not having clear and harmonised standards for influencer advertising, and Section 7 concludes the paper.

## Government Advertising and Social Media Influencers

Just as traders and companies have been using advertising to increase their customer base and revenue, governments have been seeking to popularise their policies through the same means. Whether called government advertising (Clarkson & Tollison, 1979; Di Tella & Franceschelli, 2011; Rose, 2003), public goods advertising, social or political marketing, public-information campaigns, public relations, issue advertising (Rutherford, 2000), or even propaganda (Dai & Luqiu, 2020), the sharing of information with the public of a country can take many shapes. According to Rutherford, the concept of “public goods” is “derived from the discipline of economics where these commodities are usually given a negative definition to emphasise their contrast to what is normal, namely private goods such as chocolate bars or automobiles” (Rutherford, 2000, p. 5). He gives examples such as “a drug-free America, social justice and public health, a united Canada or a sovereign Quebec, economic progress, unspoiled nature, world peace, crime-free streets, and on and on” (Rutherford, 2000, p. 5). These are examples of societal goals that are supposed to be enjoyed by the majority of the members of a society. Some of the examples might come across as universally beneficial to society as a whole, while some others may entail specific policies that are open to politicisation—maybe a drug-free America is not a goal shared by all members of society. This remains an issue when claiming that government information is neutral, which is embedded in the definition of government advertising: “necessary government information campaigns which are neutral in nature and not liable to be perceived as creating a partisan benefit for the ruling party” (Young, 2006). The political subjectivity of government information is a relic of the propaganda phenomenon as experienced in the Second World War, used as “lies and lying, the misinformation the enemy manufactured to persuade its victims and the unwary” (Rutherford, 2000, p. 7). This propaganda model has adapted to new socio-economic and geopolitical realities, and it can be argued that some state messaging may currently reproduce dominant ideologies and structures, which may perpetuate or even increase class inequalities. However, another perspective is equally important: In the era of evidence-based policy making (Nicolosi et al., 2022; Pflücke, 2024), it can also be argued that some government messaging lends itself to less politicisation than was the case decades ago and that this messaging might actually improve class inequalities. If state-of-the-art science has proven that there is a causal link between smoking and cancer (US Department of Health & Human Services, 2004), educating people about this reality can help save lives, and such a policy goal can justify a paternalistic approach towards individual agency in government information, particularly if it impacts public policies such as healthcare and public spending.

Government advertising has adapted to shifting technologies and audiences. In 1965, that meant using television for dissemination, while most recently, the government has turned its attention to social media. On social media, governments can buy advertising, although more research is needed to have a good understanding of how often this happens, and which countries are more likely to be up to date with social media marketing strategies. But in asynchronous timelines and For You Pages where you can skip the otherwise dreaded television publicity timeslots, advertising can go by unnoticed. Even worse, it can lead to public scandals around issues such as microtargeting, as has been the case with the European Commission trying to popularise its regulation on detecting child sexual abuse material online (Tar, 2024). Against this background, and to keep pace with the latest marketing trends, governments have turned to influencers. Put simply, influencers are perceived to be social media personalities with substantial clout online. They can be “micro-celebrities” within a niche expertise and audience but also mega-popular Internet famous people (Hudders et al., 2021). They can have public individual or institutional identities or be completely faceless. Legal definitions have mainly linked influencers to their commerciality (BEUC, 2023; Michaelsen et al., 2022). Their success as commercial actors is intrinsically linked to the parasocial relationships between influencers and their audiences (Chang Bi & Zhang, 2023). These are trust-based, unilateral relationships prompted by how audiences perceive influencers to be likable, inspiring, entertaining, and knowledgeable about a certain topic (Michaelsen et al., 2022, p. 9). Influencers are commonly engaged in commercial advertising practices, utilising their perceived trustworthiness and relatability to promote goods or services to consumers on social media through various business models (De Gregorio & Goanta, 2022; Goanta & Wildhaber, 2019). As such, the feeling of trust and expertise fostered by this relationship stems from the individual connection one feels with the respective influencer. Many influencers have political opinions and views that make them perfect vessels for government influence. According to Riedl et al. (2021), political influencers are “content creators that endorse a political position, social cause, or candidate through media that they produce and/or share on a given social media platform.” When contracting with the government, influencers receive money to share communications in the public interest. Found at the intersection of the market and the state, such transactions are difficult to legally qualify, and the resulting regulatory gap jeopardises the much fought-for transparency in advertising. In the next section, we focus on two European legal instruments to explain this regulatory gap.

## The Legal Qualification of Government Influencers

Influencers promoting goods and services to users on social media platforms have been in the spotlight for some time, particularly given the hidden or deceptive nature of influencer marketing (Bladow, 2017; Boerman & Reijmersdal, 2020; Vrontis et al., 2021). In response, national and supranational legislators have been reacting to a perceived need for more targeted and clear regulation establishing disclosure obligations (Hund, 2024). However, the same can not be said for interactions between governments and influencers. As early as 2015, the White House understood the power of social media content creators to spread information to new demographics through new media, as Barack Obama welcomed YouTube’s top creators at the time (Hank Green, Bethany Mota and GloZell Green) to interview him “about the issues they – and their audiences – care most about” (White House, 2015). In 2024, the White House hosted a series of events dedicated to the creator economy, with a much broader audience in attendance, reflecting the magnitude of the interest in the topic (Klippenstein & Boguslaw, 2024). Similarly, the Kremlin used influencers to share propaganda about its own position in the invasion of Ukraine (Chayka, 2022), and many other countries around the world relied on influencers to communicate public health advice during the Covid pandemic (Quekel, 2020).

When hired by the government, influencers engage in commercial dealings, yet the content of these dealings is political or social. This overlap leads to some concerns relating to the legal qualification of government influencer marketing under current legislation. This section examines how EU law tackles government influencers, by focusing on the UCPD and the PAR, due to the fact that they directly address advertising. Although more EU instruments pertaining to the emerging EU digital *acquis* may be of importance for this discussion, these two instruments can be considered not only as the *lex specialis* that would apply to advertising but also consider the economic practices themselves, and are not merely focused on platform architecture or platforms as techno-social systems. Moreover, our selection reflects the dual nature of government influencers, as expressed through both commerce and politics.

Looking at the UCPD, the monetisation of influencer speech can generally be a form of commercial practice if undertaken by a trader (Luzak & Goanta, 2022). Commercial practices are defined by the UCPD as “any act, omission, course of conduct or representation, commercial communication including advertising and marketing, by a trader, directly connected with the promotion, sale or supply of a product to consumers” (Art. 2(d) UCPD). To the extent that an influencer is considered a trader, meaning that they have performed monetisation activities with a certain consistency, any advertising done by the influencer online must abide by the UCPD’s disclosure standards, whether in the Annex (Luzak & Goanta, 2022), or in its general unfairness tests. Misleading advertising happens when influencers do not disclose that their posts are paid promotions, thereby hiding the commercial nature of the endorsement from their followers (Annex I, Sect. 11). Similarly, more transparency is needed when influencers falsely claim to be independent consumers rather than representatives of a trade or business, which is also prohibited (Annex I, Sect. 22). These regulations ensure that consumers are not deceived and can trust the authenticity of the information provided, thereby upholding market integrity and consumer trust. When applied to influencer marketing, the UCPD requires that influencers follow stringent standards to maintain transparency and integrity in their promotional content. Influencers and brands must disclose sponsored content, ensuring that promotional posts are unmistakably identified as advertisements. Although the UCPD predated the rise of social media as an industry, these interpretations, as well as its general application to influencer marketing, have been embraced by the European Commission (UCPD Guidelines, 2021), national courts (German Federal Court 2021), as well as legal academia at large (Goanta & Ranchordas. 2020). However, this is only the case if influencers are traders engaging in commercial practices. As has been pointed out, political messaging cannot be qualified as a service or a good that influencers promote, which makes it difficult to argue that the UCPD applies to government advertising.

Turning to political advertising, PAR is an emerging framework aimed at harmonising rules on political advertising at EU level, with some relevance for government advertising through influencers. The Regulation acknowledges in its preamble that “[p]olitical advertising can take many forms, including paid content, sponsored search results, paid targeted messages, promotion in rankings, promotion of something or someone integrated into content, such as product placement, influencers and other endorsements” (PAR Preamble, para. 1). According to Article 2(1), the Regulation applies to political advertisements disseminated in the EU or directed at Union citizens, regardless of the provider’s location. Article 3(2) further elaborates on what “political advertising” means—namely “the preparation, placement, promotion, publication, delivery or dissemination, by any means, of a message, normally provided for remuneration or through in-house activities or as part of a political advertising campaign.” This message can belong to “ a political actor” or be “liable and designed to influence the outcome of an election or referendum, voting behaviour or a legislative or regulatory process, at Union, national, regional or local level.” At first sight, although it is debatable whether governments and their agencies are political actors because the “political” element plays a different role in elections than in the technocratic structures making up the government, it may seem this definition could potentially cover government influencers. However, Article 3(2) also lists the types of communication that are not included in the definition of political advertising. One of these exceptions refers to “public communication that aims to provide official information to the public by, for or on behalf of any public authority of a Member State or by, for or on behalf of the Union, including by, for or on behalf of members of the government of a Member State, provided that they are not liable and designed to influence the outcome of an election or referendum, voting behaviour or a legislative or regulatory process.” “Official information” is not further defined, but the context in which this concept is used seems to relate, among others, to the formal capacity of the government, its members (e.g. ministers and ministries), or public authorities (e.g. agencies). The interpretational issues arising from these definitions are too restrictive to argue that government influencers would generally fall under their ambit. As a result, while political advertising by influencers in relation to elections and parties may very well be governed by the Regulation, public information campaigns undertaken for the government are most likely not.

Since the UCPD does not cover political communications, and the PAR does not cover official information to the public by the government, a grey area takes shape around government influencer marketing. This can have concerning effects on consumers and citizens alike. As an illustration, China and Russia have been using advertorials to natively promote news stories from government-controlled media outlets, deemed by Dai and Luqiu (2020) “camouflaged propaganda.” As can be seen in both regimes described above, transparency—particularly expressed through disclosures—is the central solution for the dishonesty problem of hidden advertising. However, even when obligations to disclose are clear and mandatory, as for commercial influencer marketing, in practice, disclosures are still very rarely done, and authenticity performance as hidden advertising remains a plague for transparency standards (Ershov & Mitchell, 2020; Mathur et al., 2018). This is worsened by grey legal areas that complicate both consumers’ and advertisers’ perception of the adequate level of disclosure.

In the absence of statutory disclosure rules for government influencers, we can turn to their contracts with the government as private regulation. In the next section, we introduce an empirical assessment of influencer contracts concluded with the Dutch government to inquire whether contractual practices provide private, voluntary solutions for the transparency dilemma, or whether additional transparency measures are necessary.

## Government Influencers in the Netherlands

We focus on the Netherlands as a jurisdiction where a lot of news of influencer campaigns have come to light in the past years (Boender, 2024; NOS, 2024; van Leeuwen, 2020), showing an increased interest of the government in influencer marketing. We explore specific examples of contractual interactions between the Dutch government and influencers, with the aim to (i) identify and understand the characteristics of the commercial dealings between the government and the influencers in their service and (ii) analyse the contractual practices around these dealings.

### Data Collection, Dataset, and Methodology

To understand how influencers produce government communication in the Netherlands, we examine FoI requests on government influencer campaigns from the Dutch government between 2020 and 2024. As we demonstrate, FoI requests provide an avenue to collect data on the use of influencers by governments, including contracts. In the Netherlands, FoI requests are governed by the “Wet openbaarheid bestuur” (WOB) (Open Governance Act), which was replaced by the “Wet open overheid” (WOO) (Open Government Act) in 2020.

Using the government archive, we searched for the term “influencers” and the document type: “Wob-verzoek OR Woo-besluit” (WOB request OR WOO decision), which generated 15 results. The term “content creator” was also used for search, but did not generate any results. We created a dataset comprising 10 results, removing one decision that only contained three references to influencers across 189 pages of documents, and four decisions that only contained letters but no additional documents. For example, the letter from the Ministry of General Affairs stated the ministry does not use “influencers”. We cross-checked our dataset with the archive of requests before 2020 and requests pertaining to COVID and tested other keywords such as “contentsamenwerking” (content partnership), “branded content,” and “mediapartners” to ensure our dataset included all relevant requests. 2 of the 10 requests were filed by a member of the research team.

Table 1 provides an overview of our dataset of 1302 pages of documentation from ten requests across eight Dutch government ministries. We conducted qualitative content analysis (Hsieh & Shannon, 2005; Schreier, 2014) to describe and interpret meaning in the materials. Our first step was to categorise the documents through an iterative process within the research team and we identified the following types: budget, communication (e.g. email, WhatsApp messages), contract, offer, campaign materials (e.g. evaluation reports, planning documents, presentations, campaign pitches). The textual data were then analysed through a process of systematic inductive coding and identification of patterns guided by two different areas of focus. We looked at the conceptualisation of influencers and strategic uses by governments in campaign materials and where relevant budget and communication documents. In addition, we examined characteristics of contractual agreement using contract and offer documents, which we discuss in “Government Influencers in the Netherlands.”

**Table 1:** Dataset of WOO and WOB requests on government influencer campaigns in the Netherlands

Ministry	Date	Nr of pages	Document name	Nr of influencer contracts
Public Health, Welfare and Sport	9-4-2020	28	1	1
Defence	25-7-2023	40	6	3
Social Affairs and Employment	13-9-2023	19	8	n/a
Justice and Security	2-10-2023	22	9	n/a
Infrastructure and Water Management	26-10-2023	91	10	n/a
Public Health, Welfare and Sport	30-10-2023	137	11	1
Education, Culture and Science	30-11-2023	44	12	n/a
Finance	8-12-2023	243	1	2
Interior and Kingdom Relations	26-1-2024	148	0	n/a
Interior and Kingdom Relations	22-2-204	530	23	n/a
Total		1302	79	

*n/a* not available

### The Conceptualisation of Influencers by Dutch Ministries

Drawing on our dataset of WOO and WOB requests on government influencer campaigns, we examined the selection of influencers and understanding(s) of what an influencer is within the documentation. Through a close reading of the documents, we identified 109 influencers of interest to the Dutch government. However, not all ministries nor documents identified influencers by name. For example, in the budget for the VWS PUUR Rookvrij Mei-Jul 2022, the cost for the content from the agency “We are First” BV lists the names of four influencers, whereas the cost for the content from the agency FamilyBlend refers to “7 × micro influencer (10–20 K vloggers).” We supplemented our influencer list by identifying influencers through their social media content, using campaign hashtags listed in documents (*n* = 15). The inclusion of these influencers aids our understanding of how authorities approach the selection and use of influencers. For example, by searching the hashtag #puurrookvrij on Instagram, we found posts from six additional accounts. This group also used #puurrookvrijxfamilyblend, unlike the content available for the four named in the budget, which further affirms their involvement in the campaign as micro-influencers.

In Table 2, we show the number of influencers selected for campaigns and whose content remains visible on their social media accounts. As of 1 May 2024, branded content from 70.5% of influencers used in government influencer campaigns could be accessed.

**Table 2:** Overview of number of influencers connected to Dutch government campaigns

Ministry	Nr. of influencers	Nr. of influencers used in campaign	Nr. of influencers whose campaign content is visible on their account
Interior and Kingdom Relations*	39	23	18
Defense	22	22	13
Finance	5	5	4
Infrastructure and Water Management	28	20	17
Justice and Security	1	1	1
Education, Culture and Science	7	7	3
Social Affairs and Employment	1	1	0
Health, Welfare and Sport*	33	33	23
Total	136	112	79

*Two WOO requests

We also found the same influencers were referred to and used in government influencer campaigns across ministries (see Table 5). For instance, Qucee was selected for campaigns by Binnenlandse Zaken en Koninkrijksrelaties, Defensie and Volksgezondheid, Welzijn en Sport, indicating how government communication is part of his portfolio of branded content and monetisation.

We next unpack how the list of influencers reveals a broad conceptualisation of what constitutes this type of commercial actor. Of the 112 influencers used in campaigns, we argue that only 75 fall within the definition of an influencer. We define influencers as content creators who cultivate a sense of closeness with followers and narrate their personal lives (Abidin, 2016) while engaging with commercial actors through various monetisation models (Goanta & Ranchordás, 2020). Critically, their visibility and “fame” emerge from their internet-native popularity. We, therefore, distinguish “influencers” (*n* = 75) from other actors that are part of the creative and cultural sector (*n* = 29) and other types of digital content production (*n* = 7) (see Table 3). The inclusion of other individuals such as musicians, TV presenters, and podcasters as well as organisations such as bands, blogs, and podcasts in the broad category of influencer resonates with what Bishop conceptualises as “influencer creep” (Bishop, 2025). Bishop demonstrates how the microcelebrity promotional practices of self-branding, optimisation, and the performance of authenticity originating in influencer culture “creep” into other forms of work and are enacted by other workers. The labelling of other actors as influencers speaks to how the practice of producing branded content for third parties, including government ministries, expands beyond “influencers,” indicating how the term influencer becomes a signifier for a public-facing social media account that reaches a target audience. In addition, it indicates how government agencies view influencers as any other medium (see Table 3) in that they are grouped in the same way.

**Table 3:** Classification of non-influencer actors that are labelled influencers in requests

Creative and cultural sector	Actor	1
	Athlete	5
	Band	5
	Musician	12
	Photographer	1
	Record label	1
	Spoken Word Artist	1
	TV Presenter	3
Other forms of digital content	Blog	1
	Magazine	2
	Meme account	1
	Podcast	3
Cannot find		2

Within the documents, this broad understanding of influencers is further evident. For example, in a budget overview of the Ministry of Defence’s social media campaigns, has a column labelled “naam influencer” (influencer name) yet the rows include any individual or organisation involved in the production of content, such as athletes, a band, a record label, and a TV presenter. The potential interchangeability of actors producing and sharing social media content with influencers is suggested in the WOO response letter from the Ministry of Social Affairs and Employment. The letter explains that the accompanying documents are related to the hiring of one influencer, but this term does not seem to be used in any of the documents. For example, in a Word document entitled “RI&E tijdens de Ondernemer Radio op 7 juni,” Tom Coronel is introduced as a “co-host of the radio show.” It is thus revealing, given that the WOO response is based solely on the relationship with Tom Coronel, that the ministry to some extent classifies Tom as an “influencer.” The influencer classification by the agency seems to rest upon their work across different creative sectors, which becomes coupled with their social media presence to hint at how they hold “influence.” This is perhaps most explicit in the description of Touzani in which he is identified as a “role model”:

Touzani, who once even kicked a ball with the famous footballer Ronaldinho, has more than a million subscribers worldwide on his YouTube channel and is an important role model for young people. He now uses this reach to inspire his young supporters.

Similar to the reference to Touzani’s YouTube channel size, we see within our dataset how “influence” is measured through metrics. In a slideshow “Preloved Fashion Fair Influencers” for the Ministry of Infrastructuur en Waterstaat, each influencer is introduced through a slide that establishes who the influencer is and how they relate to the target group, their number of Instagram followers, and a screenshot of their Instagram account. While the agency may have wished to avoid repetition by defining each as an influencer, it is striking that only two of the six are assigned this label. The remaining influencers are described as having a “platform,” a “meme account,” producing a “blog,” and enjoying upcycling. The description of the latter two, in particular, can be understood given the size of their respective Instagram accounts: 4143 and 6280 followers. Their inclusion indicates an understanding of different influencer types, including nano-influencers (under 10 000 followers).

Across the dataset of influencers, Table 4 shows the spread of influencers (as we define them) used in campaigns by ministries by size classification. The use of nano and micro influencers suggests a prioritisation of reaching niche audiences over a broad target group in campaigns following research that indicates endorsements by micro influencers may be more persuasive than macro (see Park et al., 2021).

**Table 4:** Size classification of influencers (according to our definition) used in campaigns (The data in this table are based on the influencer’s Instagram follower count retrieved on 1 May 2024 rather than time of selection for the campaign. Of the 75 influencers in Table 3, WOO request documents contained number of followers for 22. For this group, we calculated their classification using the follower count in the documents and found that despite both increases and decreases in follower counts, their assigned category was the same as the 1 May 2024 classification)

*Ministry*	Nano (under 10k)	Micro (10k-100k)	Macro (100k- 1mil)	Mega (1 mil +)
Interior and Kingdom Relations		6	9	1
Defense		6	6	1
Finance		2	2	
Infrastructure and Water Management	2	4	3	
Justice and Security		1		
Education, Culture and Science		3	2	1
Public Health, Welfare and Sport	1	13	10	1
**Total**	3	**35**	**33**	**4**

Overall, this shows how the government’s understanding of influencers is very broad, which could pose an issue of personal scope for law and policy that aims to cover their practices.

### How Value Is Attributed to Influencers

We also examined how documents assign value to influencers. By this we mean, how do actors (e.g. ministries, agencies) evaluate influencers as an object with worth for government campaigns and what properties are attributed to influencers. Within materials from agencies, influencers are positioned as effective channel through which to reach citizens. As the agency, Initiative, put it in their presentation for the “Publiekscampagne corona” refers to influencers are a “powerful tool,” resonating with Cheil and HPT’s presentation for “Vuurwerkcampgne 2022” that influencers “play a major role in providing information to young people” and setting trends. As this quote exemplifies, the identified target group of campaigns using influencers is primarily young people. At times, this is supported by statistics showing the rates of social media use by young people to reinforce the value of reaching this group as followers of influencers. As such, we see the terminology of “followers” being adopted, rather than citizens or audiences, bringing with it the framework of influencer culture. The following extract from the Ad Alliance’s evaluation presentation on the Gemeenteraadsverkiezingen campaign illustrates how the construction of “young people and voters” is also tied to their relationship with influencers (Supergraande is the name of a podcast hosted by two influencers):

Develop a campaign in which Young people and voters with a migration background are reached, informed and motivated to vote during the municipal elections in 2022.

We have sought cooperation with Supergaande. For this purpose, Supergaande creates the format Supergaande Helpt, where Supergaande helps their followers with problems they encounter.

In this example, we also see that the objectives of the campaign are in relation to voters with a migration background, reinforcing the value of using influencers to reach specific niches.

The value that influencers bring is not only in their access to target groups but also in their method of communication, which is positioned as entertaining and authentic by agencies. This emerges in how influencers as a marketing approach are presented in general and how specific influencers are promoted to agencies or instructed to produce content for the campaign purpose:

By using influencers we create attention in an interactive and authentic way. The influencers appeal to the target group of Millenials and Generation Z who prefer not to be educated by standard advertising communications. Influencers create appealing content that the target group really wants to look at and by entering into collaboration with influencers, a message can be conveyed faster and in an original way. (Document 15)

This influencer makes the *educational aspect of the campaign fun*, for example by sharing a personal experience and working with questions/question stickers to involve followers in the subject.

In general, we think it is important that the entertainment content remains high and fits with Touzani TV. It should not become an MDT explanation video. Especially in sections 2 and 8, the emphasis will literally be on MDT, but otherwise we mainly want to radiate the fun of it and other MDT people have their say!

Provide authentic and relevant content that matches the style of your Instagram channel and personality. Convey the message of Money Week and the theme ‘Control the bling’ as best as possible.

For us, you are the right person to make people more aware of this in a fun and light-hearted way.

Collectively, these extracts reveal an expectation that influencers can integrate public value messages into their fun, entertaining content in ways that will be relatable and authentic for young people. As scholars have demonstrated (Abidin, 2015; Abidin, 2018; Duffy, 2017; Marwick, 2013), such strategies of self-presentation and performance are central to the labour of influencers and their production of value. On the one hand, then, agencies showcase how government ministries can capitalise upon the phenomena of influencers by highlighting the value that using influencers can bring in creating branded content for government ministries. The “possible approach” exemplifies how influencers blend their personal stories (in this case, playing with fireworks) with public service message (how to do this safely).

There is also a disconnect between their (potential) value and use. For example, the diversity and inclusion campaign for the Ministry of Defence involved interviews with Queer people at the Defence Department. Notwithstanding the performances of authenticity and relatability in the content itself, we wish to point to the decision to restrict influencers to a particular format (interview) and use another outlet (Winq Magazine social media channels) to disseminate content does not embrace the full potential of how influencers could be utilised in such a campaign. It is instead reflective of an approach adopted by other ministries in our dataset in which the value of the “influencer” seems tied to their recognisibility and as such, their involvement is oriented towards their presence in the filming of content or production of music.

More generally, we observe through agency presentations and emails in the WOO request documents, the involvement of agencies and ministries in shaping the message integrated into content through suggested formats, scripts, feedback, review and approval processes. While issues of brand safety and the alignment of the brand with content are part of influencer marketing in general, we emphasise in the context of influencers being used in campaigns for ministries how this impacts the agency of influencers in the production of original content and at times, influences their freedom of expression given the political nature of some messaging. This tension is showcased through the following selection from a list of “conditions” provided by Initiative for the “Provinciale staten en waterschapsverkiezingen” campaign:

- The content should stay away from political colour and prioritizing social issues;
- The content should stay away from major polarising themes;
- When we use role models, it is important that they have a positive attitude towards voting and have the intention to vote now;
- Please note: This is a central government campaign. That means it is sensitive. It is extremely important that the Ministry of the Interior and Kingdom Relations has enough room to provide feedback without affecting the authenticity of the content.

### Content Range of Government Influencer Campaigns

Based on our identification of content as we outlined in “The Conceptualisation of Influencers by Dutch Ministries”, we now examine how influencers shared across platforms and disclosed this content as government communication. Table 6 (Annex) provides an overview of the content shared by 75 influencers that is visible across the identified platforms. While this does not provide a full picture of content produced for each of the listed campaigns, it gestures towards different approaches to using influencers. For example, the Ministry of Defence’s “Govert in het leger” campaign centres on one influencer generating 10 YouTube videos and 19 Instagram posts compared with the Ministry of Binnenlandse Zaken en Koninkrijksrelatie’s DigiD campaigns and Volksgezondheid, Welzijn en Sport’s #alleensammen campaign in which each influencer shared only one Instagram post.

This table indicates that Instagram was the preferred platform followed by YouTube and TikTok. Some of the content has the same or very similar captions as well as videos indicating posting cross-platform, which may lead to small differences between the disclosure of the paid partnership. For example, the YouTube Shorts post includes the paid partnership tag (upper left corner) and the equivalent toggle is not used for the Instagram post. Similarly, the ParraTV TikTok post has no disclosure in the caption, unlike the Instagram post which uses #ad at the end of the caption.

**Fig. 1:**
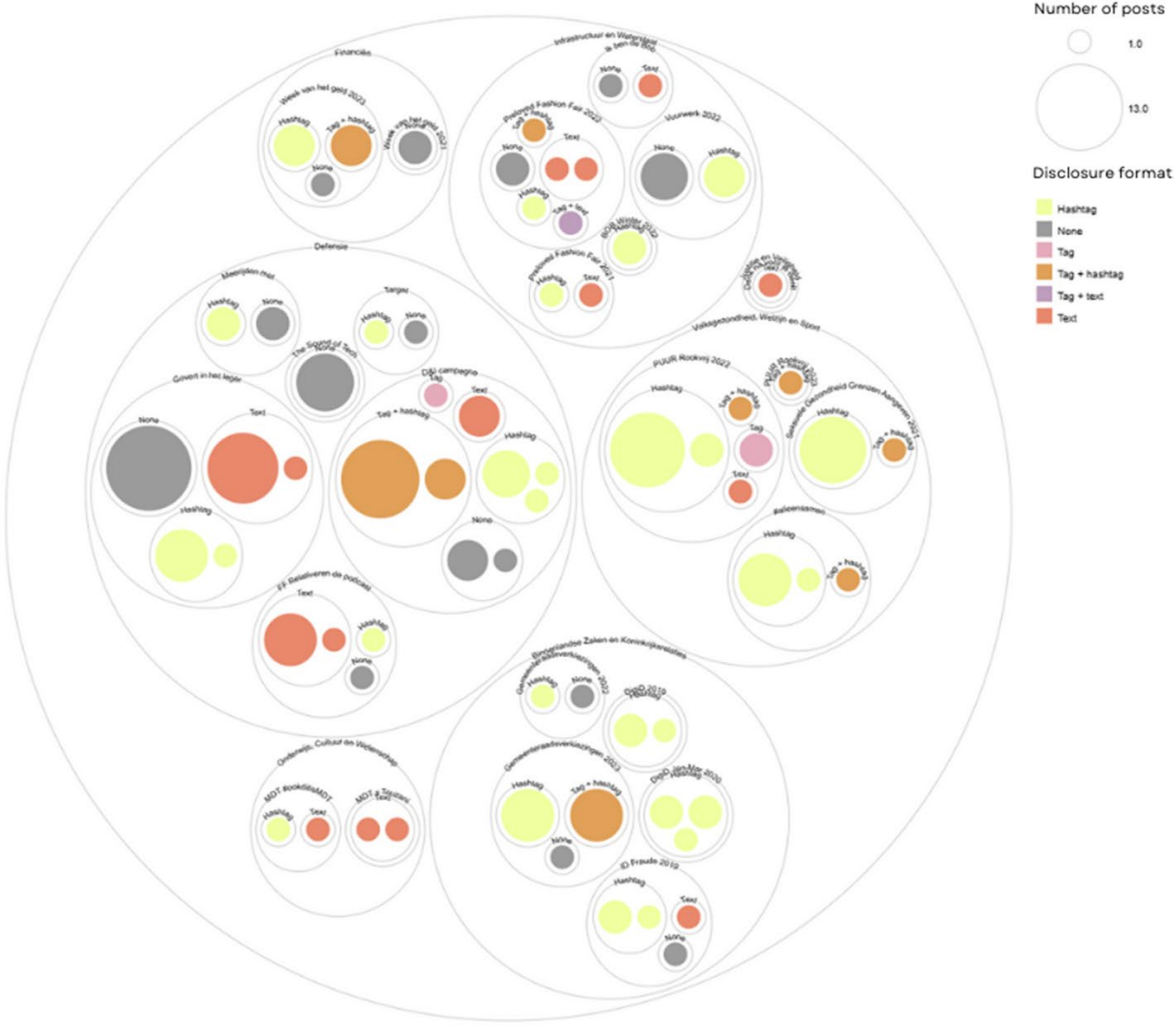
Forms of disclosure in influencer government campaign content organised by ministry, campaign and format

Looking at the dataset as a whole further illuminates differences in disclosure, which vary between and within ministries, campaigns, and platforms (see Figure 1 and Table 7 in Annex). While the most common form of disclosure is #ad (*n* = 41), this is followed by captions and titles that lack any form of disclosure (*n* = 38). Nineteen posts used the paid partnership tag, which included only one TikTok video and none from Snapchat despite the availability of this platform feature. If we look at individual influencers across and within platforms, there are differences in their posts in how they disclose their partnership as exemplified in Table 8. Such inconsistency indicates a potential lack of clarity in what form disclosures should take and an absence of standardisation across platforms.

## The Private Regulation of Government Influencers

In this section of the paper, we analyse the relationship between influencers and the Dutch government using the contracts we collected. Although they are a fundamental source of rights and obligations for parties, given the bilateral nature of such agreements, influencer contracts are not often examined in relevant scholarship. With the FoI, we were able to extract seven contracts as seen in Table 1. We focus on contracts as the instruments of private governance that show existing practices around transparency, as well generally how power is distributed across the contractual relationship. The purpose of this part is not to provide a systematic analysis of the contractual text but to exemplify some instances relevant for our analysis. We investigate the seven contracts by looking at six types of clauses:

- Parties: this clause showcases the relevant stakeholders and helps us give shape to the government advertising supply chain.
- Disclosures: this clause (wherever present) indicates the parties’ take on awareness and importance of transparently disclosing the commercial transaction behind the posts.
- Non-disparagement clauses: this clause establishes an obligation primarily of the influencer to not contradict the information in the campaign or put negative light on the government.
- Control over content: this clause reveals whether and to what extent there is creative freedom, on the one hand, or a need for approval from the government for campaign content.
- Exclusivity: this clause indicates whether influencers can be restrained from engaging in other transactions related to or at the same time with the government campaign.

### Parties

Three types of contracts emerge out of the seven contracts under analysis. First, there are contracts concluded between advertising and talent agencies. Given the framework agreements, the government is consistently represented in these contracts by an ad agency that deals on its behalf. An example is the contract and standard terms accompanying Document 1, concluded between an ad agency (Roorda), representing the Ministry of Health, and a talent agency (SPEC Entertainment). While the Ministry of Health was not explicitly party to the agreements, it is referred to several times in the contract and terms. However, while officially only Roorda and SPEC Entertainment are parties to the agreement, it was, in fact, also signed by the influencers represented by SPEC Entertainment. Another example is the contract accompanying Document 11, also concluded between the Roorda and a talent agency (Noah’s Ark). Second, there are some contracts concluded between talent agencies and influencers. These contracts are the two contracts in Document 6. One of them also lists the Ministry of Defence as a party to the contract, besides the talent agency (Influencers of Sports) and the influencers. Third, there are some contracts concluded between ministries and talent agencies (e.g. Document 13, contract 1 concluded between the Ministry of Finance and talent agency Next Chapter; contract 2 concluded between the Directorate of Financial Markets and ad agency We Are Era).

Besides the question of who the parties are, there is also the question of the capacity in which the parties are acting. This is particularly important for some of the influencers. When engaging in consistent monetisation, influencers are perceived to be professional parties (e.g. traders under the UCPD). The more contracts and revenue they obtain, the more likely it is for them to be incorporated, or at least act as registered freelancers. In some of the contracts in our dataset, it seems that to not always be the case. In the standard terms of Document 1, for instance, we see that Article 1 refers to the influencers as “natural persons,” indicating that the artists are operating in a personal instead of a business capacity. While this may not matter for the content itself, it does for the contractual liability regime applicable to the influencer when there is a dispute, a (financial) claim, or non-compliance. The separation of legal personhood between personal and business is essentially the wall that protects the influencer’s private life from its business life. Influencer Famke Louise—who signed the Standard terms in Document 1 as a natural person—later broke the non-disparagement clause, raising the question of how she is liable in her personal capacity.

### Non-disparagement Clauses

Most contracts contain a non-disparagement clause, meant to protect parties from negative repercussions possibly arising out of their collaboration.

All parties involved will refrain from making negative and/or critical statements about each other and/or about the Client and, more generally, will refrain from actions that may damage the reputation of the other and/or of the Client and about the Client's Products. They also declare that there are no ongoing issues that could harm the Client and/or the campaign.

The Standard Terms in Document 1 stipulate that in case of a breach of the non-disparagement clause, the influencer has no right to remuneration. However, there is no stipulation of what happens if non-disparagement occurs after payment and the campaign is over. This leaves significant uncertainty as to what power the Ministry would have to order payment of a fine or reimbursement of the payment in case of a breach of the clause. The contract accompanying Document 13 also contains a non-disparagement clause. Compared to Document 1, the clause leaves the Ministry more leeway to terminate the contract and to claim damages should the influencer be found in breach. More generally, the contract allows for termination and/or damages to be paid in case the Ministry suffers reputational damage, specifically in case of run-ins with law enforcement (i.e. arrest of the influencer) and other content collaborations that contravene the objectives of the Ministry’s campaign.

### Disclosures

The contracts in our dataset do underscore the necessity for clear and consistent disclosure of sponsored content across platforms, with specific guidelines tailored to various media channels. This includes mandatory hashtags and logo elements, while the placement of these disclosures may not always be dictated, creating potential ambiguities (Document 1, Clause 6.5). The emphasis on using tools like “Branded Content Tools” and specifying the disclosure’s location in platforms like YouTube highlights the importance of visibility and legal compliance for some transactions (Document 13, Contract 1, Clause 3.4). Additionally, adherence to media laws is reinforced through standardised phrases and identifiers, ensuring transparency and accountability in sponsored messaging (Document 11). However, the communication between the parties sometimes provide context for their agreement. For instance, in a music campaign promoting mental health, the Ministry of Health and its agency specify the need for the campaign to not feel like an ad (Document 11):

Here, we rely on an authentic music video that does not feel like an advertisement.

Although Contract 1 (Document 13) does not include names of influencers for the Money Week 2023 campaign, referring instead to “the influencer,” our dataset of influencer content includes branded content produced for this specific campaign. As Table 7 indicates, of the seven Instagram posts that remain visible from the campaign, disclosures took the form of #samenwerking (*n* = 3) and #samenwerking and paid partnership tag (*n* = 3). One Instagram post did not contain any form of disclosure. Additionally, #samenwerking was at the end of the caption. Thus, despite the inclusion of clauses in the contract that outline how to correctly disclose commercial content, these results indicate how content is delivered in ways that are inconsistent.

### Control Over the Content

Looking at the division of power between the contractual parties, all contracts ensure that the ministry, sometimes via the advertising agency, retains substantial control over the content curated for the campaign by the influencer. Looking at the contracts in conjunction with the communications between the ministries and the agencies paints a picture of the government retaining control to the extent that content is more often than not scripted and subject to strict approval. In some cases, e.g. in Contract 1 of Document 13, the government set out to script the whole campaign word-for-word, leaving effectively no creative autonomy with the influencer. Furthermore, content is often redacted and altered after it is shared with the government ministry for prior approval, raising essential questions about the influencer’s creative freedoms.

Given that the government has established a lot of power over the influencer’s content through the contracts, it is the division of power that creates tensions for freedom of speech. While it is an established practice for advertisers to retain control over their messaging through influencers, it usually does not raise many concerns for freedom of expression. After all, these campaigns are primarily horizontal relationships between private parties. In horizontal relationships, parties are expected to negotiate the most optimal terms for themselves, and fundamental rights, i.e. freedom of expression, were not established to protect parties outside of vertical relationships between the individual and the state—save from extreme cases.

However, the contracts at hand concern relationships between the most powerful public actor—the state—and the influencer. While the influencer can act in a business capacity, the fact remains that a state actor, by retaining significant control over the content, limits the creative autonomy of the influencer. On the front end is the scripting of what (and what not) to say. On the back end, there is the review, redaction, and approval of the content before it may be posted. As such, the state leverages its power to dictate the influencer’s expression. Of course, the more nuanced view includes the fact that the state acts in a private law capacity when contracting with influencers. Moreover, it is reasonable that the state has room to dictate what the influencer campaign should look like. After all, they are the client paying for content that matches their view of the campaign.

### Exclusivity

One contract sets forth strict non-circumvention rules to maintain the integrity of the agency-client relationship, prohibiting the client from directly engaging with the influencer for business purposes during the contract term and for at least one year afterward. This provision ensures that all requests and communications with the influencer must go through the intermediary agency, safeguarding their role and interests in the partnership (Document 13, Contract 1, Clause 9.1).

### Discussing Empirical Findings: Power Balances in Private Governance

By analysing the parties to the contract, we note that a lot of the contracts actually refer to “talent” and employ singers or actors as more classical celebrities, but with the purpose of pursuing influencer marketing. The contracts reveal a rich supply chain with different types of agencies, such as ad, talent, or influencer agencies that mediate the influencer marketing relationship. Each of these contractual actors will have their obligations, but also rights. As we have seen, for instance, talent agencies rely on clauses that ensure their exclusivity—namely not allowing influencers and ministries to deal without them.

Our analysis generally reveals that overall, ministries are interested in using influencers and their popularity, but they also want to control such collaborations. This might arise out of a need to stay out of controversies, and other forms of negative publicity, but it might also be a sign that public authorities do not really understand the value influencers can bring to their communications. Authenticity is desired because it is what makes social media content popular right now, although on a theoretical note, the concept of authenticity is very subjective and interpretative (Sorensen & Kramer, 2024). In their work with the government, influencers are not left to their own devices to perform this authenticity with creative freedoms. Ministries seem to want to retain a lot of creative control, sometimes by even using scripts that they mandate for podcasts and live meetings.

Most importantly for our analysis, disclosure references are present in a lot of the contracts we analysed. Ministries seem to be aware of a moral (if not legal) need to disclose advertising, although this generally depends on the type of content and platform where that content is disseminated. Still, it is important to note that even public actors such as the government will try to push influencers to make content that comes across as more organic and less like advertising.

In general, the clauses we elaborate upon above show that as the client, the government retains a lot of power in dealing with influencers, particularly when imposing extensive control over the content. This might be explained through the nature of the selected influencers (e.g. very rarely if at all mega-influencers). Government agencies often cannot afford to collaborate with really big and expensive influencers. At the same time, influencers with more power may be wary to engage in contracts with the government, fearing potential retaliation from their audiences if the messaging does not perfectly align with their brand identity. The influencers who concluded contracts for the campaigns analysed in this paper are the ones who either accepted such a deal due to financial incentives, due to interest in the campaigns, or both.

## The Elephant in the Room: The Waning Scope of European Consumer Law

Our paper relies on an empirical investigation of government influencers in the Netherlands to highlight how the hybrid nature of government influencer marketing, which straddles both commercial and political realms, creates a grey area that is not adequately addressed by either consumer protection or political advertising regulation. Overall, the potential societal harm behind this example is the opacity of advertising. Government advertising may be perceived as a morally legitimate public good when those in charge of the government advertise policies that are evidence-based ways to improve society as a whole. However, even democratic governments engage in advertising as propaganda (Dai & Luqiu, 2020), and democratic backsliding, even in EU Member States is a reality that can shed a lot of concerns about the scenarios of governments working with influencers to push out messages that target vulnerable groups in society, such as women, sexual minorities, or children. Without a clear demarcation between advertising and non-advertising content, social media conflates political, commercial, and social content altogether, providing the same architecture to be used by the same voices creating the same content for the same users. The only difference is who pays for it. Currently, this difference is enough to set government advertising apart from other forms of advertising that benefit from more rigorous regulation.

Although in EU policy, advertising has been an eminently consumer law matter, the proliferation of commercial channels for political and government advertising is currently leading to gaps in the protection of consumers as the audiences of such channels. For almost two decades, the UCPD has managed to tackle complex business practices, from advertising disclosures and pyramid schemes to consumer harassment, all for the purpose of manipulating consumers into making transactional decisions they otherwise would not engage in. With the UCPD providing concrete solutions for the consumer harms that arise out of government influencer marketing, EU consumer protection is an elephant in the room when it comes to this debate: it is there in the regulatory background, but not at all acknowledged in the context at hand. There are at least two reasons why the UCPD’s regime should also extend to government influencer marketing, whereas in its current form it does not tackle this issue, as we explored above.

The first argument in this sense is that even some of the participants in this industry acknowledge that transparency should follow the rules of commercial advertising, as it has been emphasised by our empirical findings. One of the most significant findings from our analysis is the inconsistent application of disclosure standards across different contracts, as well as across written agreement and practice. While commercial influencers are generally well-versed in the use of hashtags like #ad or the use of branded content tools, our study shows that government influencer contracts often lack specific instructions on how disclosures should be made, and even when they do include such instructions, they may not always be followed in practice. This creates ambiguity and can undermine the effectiveness of transparency efforts. For instance, certain contracts require the use of specific hashtags such as #ad, #rijksoverheid, and #alleensamen but do not specify where these should be placed in the content, leaving room for interpretation and potential non-compliance (Document 1, Clause 6.5). In contrast, other contracts outline precise requirements for disclosure placement, such as the beginning of a YouTube description, to ensure compliance with media laws (Document 13, Contract 1, Clause 3.4). This discrepancy highlights a regulatory gap in how government communications are standardised. Currently, transparency is left to contractual mechanisms of private regulation, which as we have seen may not even be consistent across the same units of government. Overall, the analysed contracts were not uniform in content, structure, or obligations. We expected the government ministries, as the more powerful party in the relationships, but more importantly, as the organisation acting in the public interest, to ensure that the contractual terms would be similar in the different campaigns. After all, the Ministry of General Affairs retains significant control over government campaigns because the public must see the government as uniform and because tenders govern the relationships. This was, however, not the case in practice, and the contracts varied a lot from each other.

The second argument why the UCPD should also cover government influencer marketing is that the similarities between different types of advertising are too great to justify separate legal regimes which may lead to the overall weakening of consumer protection. This argument echoes the work of Helberger and colleagues (2021), who proposed a framework for unfair political advertising practices inspired by the UCPD. Similarly, De Gregorio and Goanta (2022) discussed the expansion of the UCPD to political influencers. These views are based on the increasing impossibility of distinguishing between different types of organic and advertising content in environments such as social media, shaped and shaping the imagined audiences who are often deprived of additional context to better analyse how to protect themselves from the dangers of content with hidden purposes (Marwick & boyd 2010). On the Instagram profile of an influencer, one post can advertise coffee, another can encourage people to vote, and another can promote financial responsibility. Absent clear advertising cues and transparency standards, consumers are left to guess what may or may not be authentic content by one of their favourite online personalities with whom they have an emotional connection. Different legal standards applicable across the pixels of the same Instagram screen require a high level of legal literacy on behalf of the influencer and their management. Yet the complexity of the resulting fragmented legal regime is often difficult to navigate even for legal experts, let alone for influencers represented by talent agencies who do not only specialise in marketing. The lack of clear standards applicable horizontally across their advertising services allows influencers to justify an overall lower standard of transparency, accountability, and consumer protection. It is expected that at least for commercial advertising, the upcoming Digital Fairness Act will amend or complement the UCPD to govern influencer marketing more explicitly. We can only hope that this will be done in a holistic manner, where the commercial activity of influencers will not be further parsed based on the nature of the content.

## Conclusion

In conclusion, this paper has explored the intricate landscape of influencer marketing regulation within governmental contexts. Beginning with an introduction to their significant influence on consumer behaviour and public opinion, we navigated through the legal framework under the UCPD and PAR, highlighting its pivotal role in mandating transparency to safeguard consumers from deceptive practices in sponsored content. The empirical study on Dutch government influencers, derived from hand-selected comprehensive FoI requests, provided valuable insights into how ministries conceptualise and strategically deploy influencers, illuminating trends such as “influencer creep” and the diverse roles they assume in public communication campaigns. Analysing contractual dynamics revealed power dynamics challenges and underscored influencers’ legal status as natural persons under consumer law, prompting actionable policy recommendations to enhance transparency and fairness in governmental engagements with influencers. Ultimately, this study underscores the critical need for adaptive regulatory frameworks to uphold ethical standards, foster transparency, and maintain public trust in influencer-driven communications across governmental and political spheres. This is particularly due to the complexity of parallel legal regimes that apply to influencer marketing in general and government influencers in particular. Although derived from commercial advertising, government influencer marketing is not as such governed by consumer protection rules. However, consumer instruments such as the UCPD should be extended in scope to apply to more forms of advertising on social media, for the purpose of increasing consumer protection.

## Data Availability

Data is provided within the manuscript or supplementary information files. The authors declare no competing interests.

## Annex

Table 5, 6, 7 and 8.

**Table 5:** Influencers mentioned in more than one ministry in WOO requests

Influencer	Nr. of ministries	Binnenlandse Zaken en Koninkrijksrelaties	Defensie	Infrastructuur en Waterstaat	Onderwijs, Cultuur en Wetenschap	Volksgezondheid, Welzijn en Sport
Qucee	3	Selected for campaign	Selected for campaign			Selected for campaign
Bonnie Nottroth	2	Not selected for campaign			Selected for campaign	
Defano Holwijn	2			Selected for campaign		Selected for campaign
Hanwe	2	Selected for campaign				Selected for campaign
Ik vrouw van jou (Joyce & Scarlet)	2	Selected for campaign				Selected for campaign
Jesse Hoefnagels	2	Not selected for campaign		Not selected for campaign		
Jiami Jongejan	2	Selected for campaign			Selected for campaign	
Nesim El Ahmadi	2	Selected for campaign	Selected for campaign			
Paulien Tilstra	2	Not selected for campaign		Not selected for campaign		
Shaquille Polak	2				Selected for campaign	Selected for campaign
Sophie Milzink	2	Selected for campaign				Selected for campaign
Thomas Brok	2				Selected for campaign	Selected for campaign

**Table 6:** Overview of content shared by influencers in government campaigns across platforms

Ministry	Campaign name	Nr. of influencers	*Platform*						
			FB	IG	Personal blog	Snap	TikTok	YT	Total
Binnenlandse Zaken en Koninkrijksrelaties	DigiD 2019	3		3					3
	DigiD Jan-Mar 2020	5		5					5
	Gemeenteraadsverkiezingen 2022	1		1				1	2
	Gemeenteraadsverkiezingen 2023	5		5		1		5	11
	ID Fraude 2019	4		3				2	5
Defensie	D&I campagne	6		12		1	9	6	28
	FF Relativeren de podcast	1		1			6	1	8
	Govert in het leger	1		19				10	29
	Meerijden met	2*		4					4
	Target	2*		2					2
	The Sound of Tech	2		4				2	6
Financiën	Week van het geld 2021	2		2					2
	Week van het geld 2023	2		7					7
Infrastructuur en Waterstaat	BOB Winter 2022	2		2					2
	Ik ben de Bob	1		1				1	2
	Preloved Fashion Fair 2021	2	1	1					2
	Preloved Fashion Fair 2022	6		7					7
	Vuurwerk 2022	6		4	3				7
Justitie en Veiligheid	Denk na voor je deelt	1		1					1
Onderwijs, Cultuur en Wetenschap	MDT #ookditisMDT	2		2					2
	MDT x Touzani	1						2	2
Volksgezondheid, Welzijn en Sport	#alleensamen	7		7					7
	PUUR Rookvrij 2022	9		16					16
	PUUR Rookvrij 2023	1		1					1
	Seksuele Gezondheid Grenzen Aangeven 2021	6		2			7		9
Total		80	1	112	3	2	22	30	170
	Govert in het leger	Instagram		1					
			#ad	5					
			#samenwerking	1					
			No disclosure	10					
			No disclosure - spinoff content	1					
			Thanks	1					
		YouTube	No disclosure	1					
			No disclosure - spinoff content	1					
			Samnwerking met	8					
	Meerijden met	Instagram	#ad	2					
			No disclosure	2					
	Target	Instagram	#ad	1					
			No disclosure	1					
	The Sound of Tech	Instagram	No disclosure	4					
		YouTube	No disclosure	2					
Financiën	Week van het geld 2021	Instagram	No disclosure	2					
	Week van het geld 2023	Instagram	#samenwerking	3					
			No disclosure	1					
			Paid partnership tag + #samenwerking	3					
Infrastructuur en Waterstaat	BOB Winter 2022	Instagram	#ad	2					
	Ik ben de Bob	Instagram	No disclosure	1					
		YouTube	Samnwerking met	1					
	Preloved Fashion Fair 2021	Facebook	#adv	1					
		Instagram	Samen met	1					
	Preloved Fashion Fair 2022	Instagram	#ad	1					
			No disclosure	2					
			Paid partnership tag + #ad	1					
			Paid partnership tag + samenwerking	1					
			Samen met	1					
			Samenwerking	1					
	Vuurwerk 2022	Instagram	#samenwerking	3					
			No disclosure	1					
		Personal blog	No disclosure	3					
Justitie en Veiligheid	Denk na voor je deelt	Instagram	Samenwerking	1					
Onderwijs, Cultuur en Wetenschap	MDT #ookditisMDT	Instagram	#ad	1					
			spon	1					
	MDT x Touzani	YouTube	ad	1					
			Thanks	1					
Volksgezondheid, Welzijn en Sport	#alleensamen	Instagram	#ad	5					
			#samenwerking	1					
			Paid partnership tag + #ad	1					
	PUUR Rookvrij 2022	Instagram	#ad	2					
			#adv	10					
			ad	1					
			Paid partnership tag	2					
			Paid partnership tag + #adv	1					
	PUUR Rookvrij 2023	Instagram	Paid partnership tag + #ad	1					
	Seksuele Gezondheid Grenzen Aangeven 2021	Instagram	#partner	1					
			Paid partnership tag + #partner	1					
		TikTok	#partner	7					

*Dutch Performante (Chahid Charrak) was used in both campaigns

**Table 7:** Overview of forms of disclosure used in government influencer campaign

*Ministry*	*Campaign*	*Platform*	*Disclosure type*	Posts
Binnenlandse Zaken en Koninkrijksrelaties	DigiD 2019	Instagram	#ad	2
			#spon	1
	DigiD Jan-Mar 2020	Instagram	#ad	2
			#collab	2
			#samenwerking	1
	Gemeenteraadsverkiezingen 2022	Instagram	#ad	1
		YouTube	No disclosure	1
	Gemeenteraadsverkiezingen 2023	Instagram	#adv	5
		Snapchat	No disclosure	1
		YouTube	Paid partnership tag + #adv	5
	ID Fraude 2019	Instagram	#ad	2
			#partner	1
		YouTube	gesponsord door het	1
			No disclosure	1
Defensie	D&I campagne	Instagram	#ad	11
			Paid partnership tag + #ad	1
		Snapchat	No disclosure	1
		TikTok	#ad	4
			No disclosure	3
			Paid partnership tag	1
			Paid partnership tag + #ad	1
		YouTube		3
			Samnwerking met	3
	FF Relativeren de podcast	Instagram	#spon	1
		TikTok	No disclosure	1
			spon	5
		YouTube	Samnwerking met	1
	Govert in het leger	Instagram		1
			#ad	5
			#samenwerking	1
			No disclosure	10
			No disclosure - spinoff content	1
			Thanks	1
		YouTube	No disclosure	1
			No disclosure - spinoff content	1
			Samnwerking met	8
	Meerijden met	Instagram	#ad	2
			No disclosure	2
	Target	Instagram	#ad	1
			No disclosure	1
	The Sound of Tech	Instagram	No disclosure	4
		YouTube	No disclosure	2
Financiën	Week van het geld 2021	Instagram	No disclosure	2
	Week van het geld 2023	Instagram	#samenwerking	3
			No disclosure	1
			Paid partnership tag + #samenwerking	3
Infrastructuur en Waterstaat	BOB Winter 2022	Instagram	#ad	2
	Ik ben de Bob	Instagram	No disclosure	1
		YouTube	Samnwerking met	1
	Preloved Fashion Fair 2021	Facebook	#adv	1
		Instagram	Samen met	1
	Preloved Fashion Fair 2022	Instagram	#ad	1
			No disclosure	2
			Paid partnership tag + #ad	1
			Paid partnership tag + samenwerking	1
			Samen met	1
			Samenwerking	1
	Vuurwerk 2022	Instagram	#samenwerking	3
			No disclosure	1
		Personal blog	No disclosure	3
Justitie en Veiligheid	Denk na voor je deelt	Instagram	Samenwerking	1
Onderwijs, Cultuur en Wetenschap	MDT #ookditisMDT	Instagram	#ad	1
			spon	1
	MDT x Touzani	YouTube	ad	1
			Thanks	1
Volksgezondheid, Welzijn en Sport	#alleensamen	Instagram	#ad	5
			#samenwerking	1
			Paid partnership tag + #ad	1
	PUUR Rookvrij 2022	Instagram	#ad	2
			#adv	10
			ad	1
			Paid partnership tag	2
			Paid partnership tag + #adv	1
	PUUR Rookvrij 2023	Instagram	Paid partnership tag + #ad	1
	Seksuele Gezondheid Grenzen Aangeven 2021	Instagram	#partner	1
			Paid partnership tag + #partner	1
		TikTok	#partner	7

**Table 8:** Different types of disclosure used by the same influencer for the same campaign

Influencer	Platform	Disclosure type	Post
Boer Ayoub (via PARRA)	Instagram	#ad	1
	TikTok	#ad	2
		Paid partnership tag + #ad	1
	YouTube	Samnwerking met	1
Defano Holwijn	Instagram	#ad	1
		#samenwerking	1
Duurzaam op kamers (Julia)	Instagram	Paid partnership tag + samenwerking	1
		Samenwerking	1
Govert Sweep	Instagram		1
		#ad	5
		#samenwerking	1
		No disclosure	10
		No disclosure - spinoff content	1
		Thanks	1
	YouTube	No disclosure	1
		No disclosure - spinoff content	1
		Samnwerking met	8
Hanwe	Instagram	#ad	1
	YouTube	gesponsord door het	1
		No disclosure	1
Ik vrouw van jou	Instagram	#collab	1
		Paid partnership tag	2
Juf Sanne	Instagram	#samenwerking	2
		No disclosure	1
		Paid partnership tag + #samenwerking	1
Touzani	YouTube	ad	1
		Thanks	1
